# Future Innovations in Novel Detection for Atrial Fibrillation (FIND-AF): pilot study of an electronic health record machine learning algorithm-guided intervention to identify undiagnosed atrial fibrillation

**DOI:** 10.1136/openhrt-2023-002447

**Published:** 2023-09-30

**Authors:** Ramesh Nadarajah, Ali Wahab, Catherine Reynolds, Keerthenan Raveendra, Deborah Askham, Richard Dawson, John Keene, Sagar Shanghavi, Gregory Y H Lip, David Hogg, Campbel Cowan, Jianhua Wu, Chris P Gale

**Affiliations:** 1 Leeds Institute for Data Analytics, University of Leeds, Leeds, UK; 2 Leeds Institute of Cardiovascular and Metabolic Medicine, University of Leeds, Leeds, UK; 3 Department of Cardiology, Leeds Teaching Hospitals NHS Trust, Leeds, UK; 4 Medical School, University of Leeds, Leeds, UK; 5 Affinity Care Primary Care Network, Bradford, UK; 6 West Leeds Primary Care Network, Leeds, UK; 7 LS25/Primary Care Network, Leeds, UK; 8 Liverpool Centre for Cardiovascular Science, University of Liverpool, Liverpool, UK; 9 Department of Clinical Medicine, Aalborg University, Aalborg, Denmark; 10 School of Computing, University of Leeds, Leeds, UK; 11 Department of Cardiology, Leeds General Infirmary, Leeds, UK; 12 Wolfson Institute of Population Health, Queen Mary University, London, UK; 13 Biostatistics Unit, University of Leeds, Leeds, UK

**Keywords:** atrial fibrillation, primary care, electronic health records

## Abstract

**Introduction:**

Atrial fibrillation (AF) is associated with a fivefold increased risk of stroke. Oral anticoagulation reduces the risk of stroke, but AF is elusive. A machine learning algorithm (Future Innovations in Novel Detection of Atrial Fibrillation (FIND-AF)) developed to predict incident AF within 6 months using data in primary care electronic health records (EHRs) could be used to guide AF screening. The objectives of the FIND-AF pilot study are to determine yields of AF during ECG monitoring across AF risk estimates and establish rates of recruitment and protocol adherence in a remote AF screening pathway.

**Methods and analysis:**

The FIND-AF Pilot is an interventional, non-randomised, single-arm, open-label study that will recruit 1955 participants aged 30 years or older, without a history of AF and eligible for oral anticoagulation, identified as higher risk and lower risk by the FIND-AF risk score from their primary care EHRs, to a period of remote ECG monitoring with a Zenicor-ECG device. The primary outcome is AF diagnosis during ECG monitoring, and secondary outcomes include recruitment rates, withdrawal rates, adherence to ECG monitoring and prescription of oral anticoagulation to participants diagnosed with AF during ECG monitoring.

**Ethics and dissemination:**

The study has ethical approval (the North West—Greater Manchester South Research Ethics Committee reference 23/NW/0180). Findings will be announced at relevant conferences and published in peer-reviewed journals in line with the Funder’s open access policy.

**Trial registration number:**

NCT05898165.

**WHAT IS ALREADY KNOWN ON THIS TOPIC**

- Population screening for atrial fibrillation (AF) guided by age or stroke risk with ECG monitoring increases AF detection rates compared with routine care and is associated with increased prescription of oral anticoagulation. However, yields of newly detected AF are low, which limits clinical effectiveness and cost-effectiveness.

**WHAT THIS STUDY ADDS**

- The Future Innovations in Novel Detection of Atrial Fibrillation (FIND-AF) pilot study investigates the use of a machine learning AF risk prediction algorithm (FIND-AF) in UK primary care electronic health records (EHRs) to guide a remote AF screening pathway, and will provide data on the yield that can be achieved with ECG monitoring across risk estimates.

**HOW THIS STUDY MIGHT AFFECT RESEARCH, PRACTICE OR POLICY**

- FIND-AF is applicable at scale in primary care EHRs. If the study demonstrates that higher predicted AF risk is associated with a high yield of AF detection on ECG monitoring, it has the potential to efficiently guide targeted early detection of AF in the community.

## Introduction

Atrial fibrillation (AF) is the most common sustained cardiac arrhythmia worldwide and confers a fivefold increased risk of stroke.^1^ It is estimated that up to 35% of AF disease burden remains undiagnosed,^2^ and 15% of strokes occur in the context of undiagnosed AF.^3^ Early detection of AF may allow the initiation of oral anticoagulation to reduce the risk of AF-related stroke.^4^

Systematic population screening for AF guided by age with or without the presence of additional stroke risk factors with a non-invasive ECG devices has been shown to be feasible, increase detection rates for AF compared with routine care, and increase initiation of oral anticoagulation. However, yields of new AF diagnosed are low at between 2.6% and 5.3%.^5–8^ Population screening of 75 and 76 years with an intermittent hand-held ECG recorder demonstrated a small net benefit in a composite outcome of ischaemic or haemorrhagic stroke, systemic embolism, bleeding leading to hospitalisation and all-cause death compared with routine care, but was limited by a yield of new of AF of only 3.0%,^8^ which hampers clinical and cost-effectiveness.^9^

A targeted screening approach towards a reliably identified subpopulation at higher risk of AF may be more effective and cost-effective. Guiding AF screening by predicted AF risk based on artificial intelligence analysis of ECGs in sinus rhythm has been demonstrated to improve yield of new AF,^10^ but ECGs are not widely available in the community which limits the scalability of this approach in the general population. By contrast, a large proportion of the population—98% in the UK—are registered in primary care with a routinely collected electronic health record (EHR).^11 12^ Thus establishing AF risk from data in primary care EHRs may be a more appropriate approach to guide population AF screening.

A previous randomised clinical trial (RCT) of intermittent non-invasive ECG monitoring compared with routine care for individuals identified as higher risk by an EHR-based risk prediction algorithm (PuLSE-AI) did not find a higher yield of AF detection from ECG monitoring.^13^ However, that algorithm had a number of shortcomings—it could only be applied to a minority of the population (35%) due to the variables it required for prediction,^14^ and it predicted 10-year AF-risk,^15^ which may not be relevant to investigating for AF in the short term.

The Future Innovations in Novel Detection of Atrial Fibrillation (FIND-AF) machine learning algorithm was developed and validated in routinely collected EHRs from over two million UK patients for prediction of incident AF within the next 6 months. It demonstrates an area under the receiver operating characteristic (AUROC) of 0.824 (95% CI 0.814 to 0.834), with robust prediction performance across both sexes and different ethnic groups.^16^ Notably, it was designed to be applicable to 100% of UK primary care EHRs.

FIND-AF was developed and validated in retrospective cohorts of patients where AF was diagnosed during routine care. The objectives of the FIND-AF pilot study are to determine yields of AF during non-invasive ECG monitoring across AF risk estimates and to establish recruitment and protocol adherence rates for a remote AF screening intervention.

## Methods and analysis

### Study design

This publication describes V2.0 of the FIND-AF pilot study protocol, dated 7 September 2023. The FIND-AF pilot study is an interventional, non-randomised, single-arm, open-label study in UK primary care.

### Study population

The study will enrol 1955 participants aged ≥30 years with a primary care EHR at general practices in the National Institute of Health and Care Research (NIHR) Clinical Research Network Yorkshire and Humber region, who do not have known AF or atrial flutter, and are eligible for oral anticoagulation.

Individuals aged ≥30 years are included because this age group were included in the development of the FIND-AF algorithm.^16^ Eligibility for oral anticoagulation is determined as men with a CHA_2_DS_2_-VASc score ≥2 or women with a CHA_2_DS_2_-VASc score ≥3.^17^ We will exclude individuals receiving any form of anticoagulation and those on the palliative care register. Eligibility for oral anticoagulation is required because the aim of the intervention is to diagnose AF in those who would be considered for anticoagulation, thereby minimising the unnecessary care, cost and anxiety for patients for whom a new diagnosis of AF would not change their management. The inclusion and exclusion criteria are summarised in box 1.

Box 1 **Inclusion and exclusion criteria**

**Inclusion criteria**

- Age at enrolment ≥30 years.
- Men with CHA_2_DS_2_-VASC≥2 and women with a CHA_2_DS_2_-VASC≥3.

**Exclusion criteria**

- Known diagnosis of atrial fibrillation or atrial flutter.
- Currently receiving anticoagulation.
- On the palliative care register.
- Unable to give written informed consent for participation in the study.
- Unable to adhere to the study requirements.

The eligible population will have their AF risk estimated using FIND-AF.^16^ Individuals will be divided into four categories based on the predicted AF risk with recruitment guided by risk strata: (1) 200 patients from the top first percentile, (2) 400 patients from the top 1st–5th percentile, (3) 500 patients from the top 5th–10th percentile and (4) 855 patients from the bottom 90th percentile. This approach aims to enrich the cohort with higher risk patients while also including lower risk patients to determine whether there is an increase in the yield of new AF diagnosis from screening across the range of FIND-AF risk estimates.^18^ Only by comparing the yield of AF detection in the higher-risk group to a lower-risk group undergoing the same ECG monitoring intervention can we assess whether FIND-AF provides additional information.

**Figure 1:**
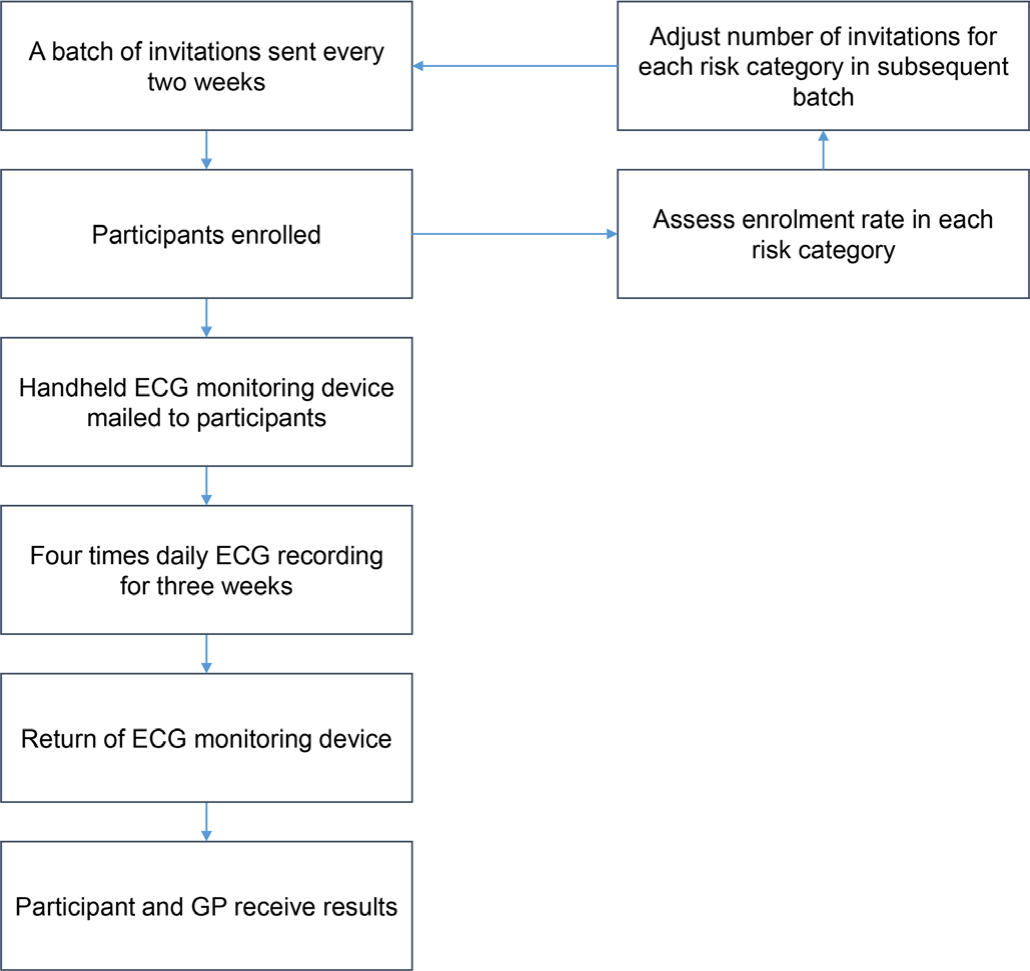
Batch enrolment, study intervention, follow-up procedures. GP, general practitioner.

Study invitations will be sent to a random sample of eligible participants in each risk category in batches until the target sample size is reached. As participants are enrolled in the study, the number of invitations for each risk category will be adjusted in the subsequent batches (figure 1).

### Enrolment method

**Figure 2:**
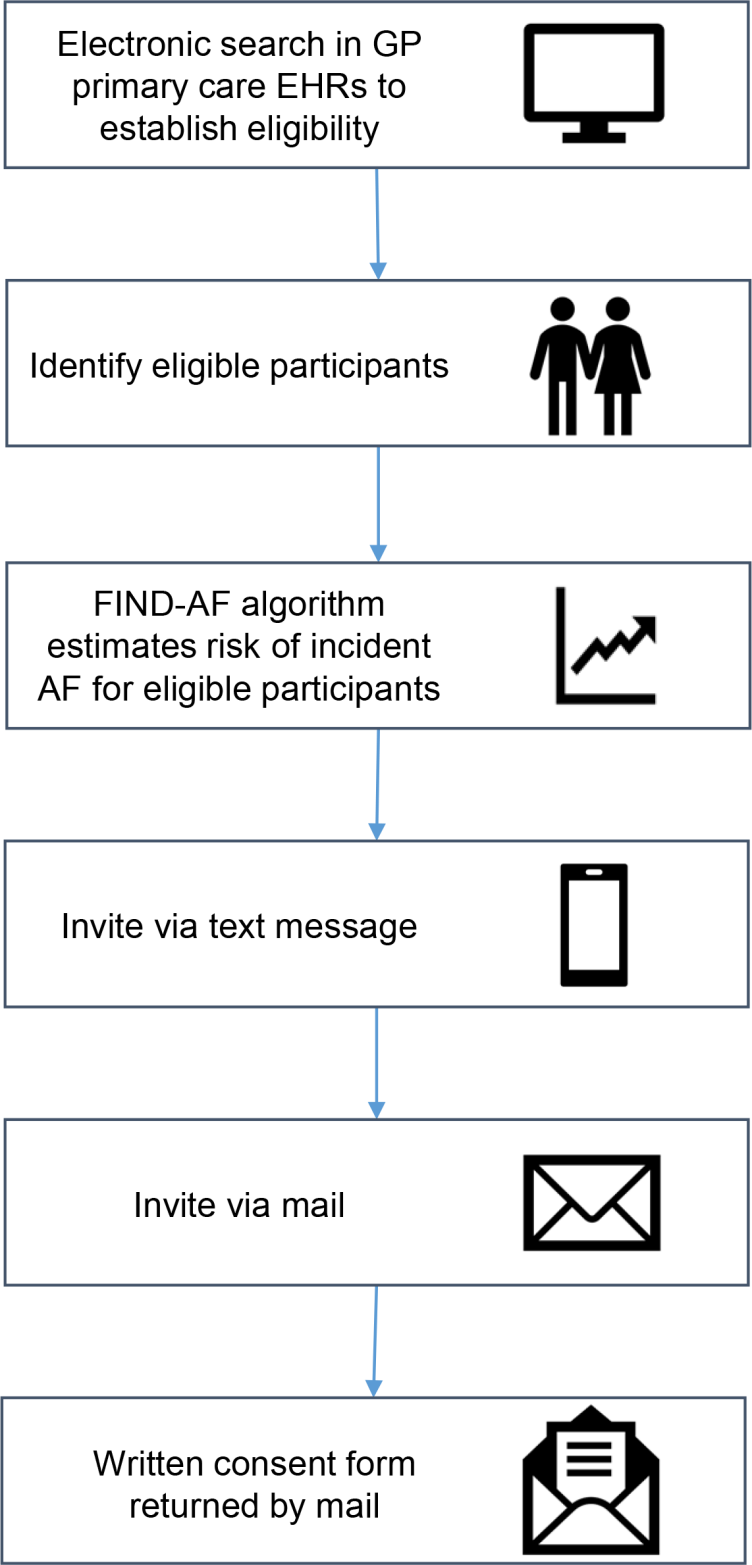
Remote consent with eligibility based on information recorded in electronic health records. EHRs, electronic health records; FIND-AF, Future Innovations in Novel Detection of Atrial Fibrillation; GP, general practitioner.

Eligible participants will be identified by the primary care team via an electronic search of general practice data (figure 2). The EHRs for potential participants will be checked to ensure they meet study inclusion and exclusion criteria. Eligibility will be confirmed by a medical practitioner who will ensure it is appropriate to contact the potential participant.

All invitations to targeted screening will occur on site by members of the primary care site team. The invitation process consists of a text message followed by an information pack in the post including a participant information sheet, consent form, data protection leaflet and free-post return envelope (figure 2). Potential participants will be supplied with a telephone number and email address to contact the study team if they wish to discuss the study and ask any questions they might have prior to providing written consent. All participants will be required to provide written informed consent by returning a completed consent form to the coordinating centre.

### Intervention

All participants will undergo non-invasive ECG monitoring (figure 1). Participants will receive a handheld Zenicor-ECG recorder via the mail with which they will be asked to record their ECG four times daily (morning, noon, afternoon and evening), or whenever they have palpitations, for 3 weeks.^19 20^ The Zenicor-ECG recorder is a single-lead ECG recorder that is CE-marked as a diagnostic device for AF.^21–23^ ECG recordings from the Zenicor-ECG are not displayed on the recorder, but will be automatically and securely transmitted digitally via a 2G mobile network to a central secure Zenicor database.

The Zenicor database has an algorithm that classifies and tags each ECG trace as ‘no tag’, ‘possible AF’ or ‘poor quality’. The algorithm has been tested in 80 149 ECGs and the negative predictive value for ECGs classified as normal is 99.9%.^24^ ECGs tagged as ‘possible AF’ will be reviewed by the research team on a weekly basis and a cardiologist will diagnose AF or any other important rhythm disturbances.

Once ECG monitoring reports have been reviewed, a standardised results letter will be sent to the participant and the general practitioner (GP). Results letters will be sent for all participants, irrespective of whether AF is diagnosed or not. The management of AF will be at the discretion of the GP, allowing doctor and participant to discuss the management strategy, including anticoagulation, independently and in line with how new cases of AF diagnosed in the community are managed in routine clinical practice. A diagnosis of AF does not require immediate action, but if there is a finding meeting the criteria for an emergent event per current clinical practice standards according to the National Institute for Health and Care Excellence, participants and appropriate clinicians will be notified. Actions will be taken following the same procedures as the established clinical workflow.

### Baseline characteristics

Baseline participant characteristics will be drawn from their primary care EHRs (box 2).

Box 2 **Participant baseline characteristics**

**Participant characteristics**

- Age.
- Sex.
- Ethnicity.

**Medical history**

- Coronary artery disease.
- Chronic kidney disease.
- Heart failure.
- Hypertension.
- Diabetes mellitus.
- Stroke/transient ischaemic attack.
- Valvular heart disease.
- CHA_2_DS_2_-VASC score.

**Medications**

- Aspirin.
- ACE inhibitor or angiotensin receptor blocker.
- Beta blocker.
- Oral anticoagulant.
- Statin.

### Outcomes

The primary outcome will be a new diagnosis of AF defined as at least one episode of completely irregular rhythm with no organised or regular atrial activity and a duration of 30 s on one-lead ECG during the Zenicor monitoring period.^20 25^ Enrolling participants at both higher and lower predicted risk of AF will provide data for the yield of AF across risk estimates, and allow the testing of the hypothesis that individuals identified at higher predicted AF risk are more likely to have AF diagnosed during ECG monitoring than individuals identified as at lower predicted AF risk.

Secondary outcomes include:

- Number (%) of people who consent to participate compared with number of people who are invited.
- Characteristics of those who consent to participate and do not consent to participate.
- Number (%) of people who consent to participate but subsequently withdraw consent or decline ECG monitoring.
- Characteristics of those who participate and those that withdraw.
- Of those who conduct ECG monitoring, the number (%) of participants who record less than 50% of the stipulated amount of ECG recordings.
- Of those who conduct ECG monitoring, the day of first detection of AF.
- Number (%) of participants who are diagnosed with other arrhythmias during ECG monitoring in participants.
- Number (%) of participants who are diagnosed with AF during ECG monitoring who then receive a prescription of oral anticoagulation within 6 months.
- Number (%) of diagnoses of AF during routine practice outside of ECG monitoring (presence of an AF diagnostic code in their primary care EHR at 6 months after enrolment).

### Sample size

Assuming 1.5% of the participants in the lower risk group have newly diagnosed AF,^2 6^ we will have 80% power to detect a significant difference if 6% of the higher risk groups have newly diagnosed AF.^10 13^

### Statistical analysis

We will calculate the incidence rate ratio of AF detection during ECG monitoring between higher predicted AF risk and lower predicted AF risk participants, using the threshold from the original development and validation paper.^16^ We will calculate positive predictive value, negative predictive value, sensitivity, specificity and AUROC for FIND-AF. We will explore different thresholds and report corresponding performance measures, which will inform whether the FIND-AF threshold to classify higher and lower risk should be altered for optimal yield.

### Patient and public involvement

The FIND-AF patient and public involvement group co-designed the study and co-drafted the consent forms and participant information sheets. Importantly, they designed the multimodal invitation (text followed by letter) to screening as they concluded that the usual invitation approach, a letter alone, may lead to poorer participation from people of minority ethnicities and lower socioeconomic classifications.^8^ The Arrhythmia Alliance will support dissemination activities.

### Limitations

The FIND-AF pilot is not an RCT. The Zenicor-ECG device is the only AF detection device that will be used in the study. Other studies have used a skin patch that can monitor the ECG rhythm continuously, for between 14 and 30 days.^10 26^ As the Zenicor device records a 30 s ECG and is being used only four times a day, it is possible that some cases of AF that would be diagnosed using continuous monitoring will be missed when using an intermittent monitoring approach. However, the Zenicor-ECG was the used for AF detection in the STROKESTOP RCT where treatment of screen-detected AF was associated with a 4% reduction in combined endpoint of ischaemic or haemorrhagic stroke, systemic embolism, bleeding leading to hospitalisation and all-cause death.^8^ Accordingly, we are reassured that treatment of AF detected during this study is clinically appropriate, whereas the optimal threshold of AF duration to be treated in continuous monitoring screen-detected AF is still to be established.^27^ This pilot study is examining AF detection through targeted AF screening, but the effect of AF screening on clinical outcomes is subject to a conflicting evidence base,^28 29^ with further RCTs ongoing.^30^

## Ethics and dissemination

The study will be performed in compliance with the articles of the Declaration of Helsinki (revised in October 2013). The study was approved by the North West—Greater Manchester South Research Ethics Committee, and the study was approved by the Health research Authority (IRAS project ID: 318197), and registered on ClinicalTrials.gov (NCT05898165). Study results will be disseminated at relevant conferences and published in peer-reviewed journals. Authorship will be decided according to ICMJE guidelines as to qualifying contributions, and the primary results manuscript jointly drafted by the co-chief investigators and the trial methodologists before circulating to remaining coauthors.

## Discussion

Primary care EHRs provide a scalable approach to guide AF screening across a nation. Hitherto, AF screening interventions with non-invasive ECG devices have been hindered by low yields of newly detected AF. This pilot study will provide data for whether higher predicted AF risk identified by the FIND-AF machine learning algorithm using primary care EHRs is associated with higher yields of AF during ECG monitoring among a population eligible for oral anticoagulation.

## Data Availability

Data are available on reasonable request.
